# Establishment of an Efficient CRISPR-Cas9-Mediated Gene Disruption System in the Lichen-Forming Fungus *Umbilicaria muhlenbergii*

**DOI:** 10.3390/jof12080622

**Published:** 2026-08-19

**Authors:** Zeyi Wang, Niuniu Wang, Haiyu Zhang, Ben Qian, Diwen Wang, Yanyan Wang

**Affiliations:** 1 State Key Laboratory of Biocontrol, School of Agriculture and Biotechnology, Sun Yat-sen University, Shenzhen 518107, China; wangzey5@mail.sysu.edu.cn (Z.W.); wangniuniu2021@126.com (N.W.); zhanghaiyu5@163.com (H.Z.); qianbenqb@foxmail.com (B.Q.); 2 Department of Botany and Plant Pathology, Purdue University, West Lafayette, IN 47907, USA; 3 State Key Laboratory of Mycology, Institute of Microbiology, Chinese Academy of Sciences, Beijing 100101, China

**Keywords:** CRISPR-Cas9, gene disruption, lichen, *UmSOM1*, morphological transition, pseudohyphae

## Abstract

Lichen-forming fungi establish intimate symbiotic associations with photosynthetic partners and play important roles in diverse ecosystems, but functional genetic studies in these organisms remain limited by the lack of efficient genome-editing tools. In this study, we established an efficient CRISPR-Cas9-mediated gene disruption system in *Umbilicaria muhlenbergii*. Using this system, we achieved the targeted disruption of six candidate transcription factors with a high replacement efficiency of up to 65.0%. No off-target mutations were detected in any of the three independent mutants examined for each target gene. Preliminary phenotypic characterization of the resulting mutants revealed that disruption of *UmSOM1* markedly impaired fungal growth, induced pseudohyphal development, and altered colony morphology and pigmentation. Compared with conventional homologous recombination, the CRISPR-Cas9 system substantially improved gene disruption efficiency, thereby overcoming a major limitation in the genetic manipulation of lichen-forming fungi. This system provides a robust platform for functional genomic studies and will accelerate investigations into the molecular mechanisms underlying fungal–algal symbiosis and morphological transitions in lichen-forming fungi.

## 1. Introduction

Lichens are unique symbiotic organisms formed by the mutualistic association between lichen-forming fungi (predominantly ascomycetes and rarely basidiomycetes) and photosynthetic partners (green algae or/and cyanobacteria) [1]. As pioneers of extreme environments, lichens profoundly drive global biogeochemical cycles and ecological succession through efficient biological weathering, carbon/nitrogen fixation, and biological soil crust formation [2,3,4]. Simultaneously, lichens produce a diverse treasure trove of secondary metabolites, playing irreplaceable roles in ecological defense, environmental bioindication, and modern pharmacology [5,6,7]. All these distinct biological traits are ultimately shaped by the unique symbiotic mechanisms inherent to the lichen association. Hence, studying the lichen symbiotic mechanism is important for the sustainable development and utilization of lichen resources; however, our current understanding of this intricate symbiosis remains fragmented [8]. With the deepening of research on the lichen microbiome, an increasing number of studies have reported that lichens harbor a diverse array of associated microorganisms. These microbes not only support host nutrition and growth through nitrogen fixation and growth promotion but also contribute to host protection by secreting antimicrobial compounds and synthesizing structurally unique secondary metabolites, acting synergistically with the host fungus to drive the ecological adaptation of the lichen holobiont [9,10,11,12,13,14]. Consequent to these findings, the classical concept of lichens, traditionally defined as a binary symbiosis between a fungus and an alga, has been revised to encompass a highly coordinated, multi-species symbiotic system [15]. However, the current understanding of the complexity and interaction mechanisms within lichens relies heavily on multi-omics inferences, and direct empirical evidence from robust experimental validation remains largely lacking.

As the only known lichen-forming fungus capable of bidirectional dimorphic transition between yeast and pseudohyphal forms, *Umbilicaria muhlenbergii* serves as an irreplaceable model for investigating the early assembly of lichen symbioses [16]. During the initial stage of symbiosis, contact with a compatible alga triggers a switch from budding yeast-like cells to pseudohyphae—chains of elongated cells that remain attached after division—which increases the contact surface with algal cells and is considered a prerequisite for establishing the symbiotic interface. This transition is co-regulated by the cyclic AMP–protein kinase A (cAMP-PKA) pathway and by Ump1, a mitogen-activated protein kinase (MAPK) orthologous to Pmk1 of *Magnaporthe oryzae* [17,18]. To date, however, only three genes have been deleted in this species: *UmGPA2* and *UmGPA3*, encoding two Gα subunits, and *UMP1*, encoding the MAPK described above. All three genes are involved in the morphological transition and symbiotic interaction, and the lack of efficient genetic tools has so far prevented functional analysis on a larger scale. The slow growth rates of lichen-forming fungi have long hindered efficient in vivo gene function studies. The application of conventional fungal genetic tools in lichen-forming fungi faces severe challenges, such as resilient cell walls that resist enzymatic digestion and extremely low protoplast regeneration rates [19]. Therefore, developing and refining efficient and stable genetic manipulation systems for lichen-forming fungi is not only essential for unveiling the molecular mechanisms of lichen interactions within a multi-species symbiotic context but also represents a critical breakthrough for deconstructing and engineering the functional traits of this complex symbiotic system through synthetic biology.

Several genetic manipulation techniques have been adapted for lichen-forming fungi. For instance, *Agrobacterium tumefaciens*-mediated transformation (ATMT) has been successfully established in *Umbilicaria* and *Cladonia* species, though it is currently restricted to random transfer DNA (T-DNA) insertion [19,20]. Recently, PEG (polyethylene glycol)-mediated homologous recombination has achieved a significant breakthrough in *U. muhlenbergii*, enabling targeted gene disruption [17]. This advancement marks a pivotal transition for lichen-forming fungal genetics from random mutagenesis to precise, gene-targeted modification.

CRISPR-Cas9 was originally described as an adaptive immune system in bacteria and archaea [21,22], which recognizes and cleaves invading nucleic acids in a sequence-specific manner [23,24]. To achieve precise genome cleavage, the CRISPR system requires two components: the Cas9 endonuclease, which provides DNA cleavage activity, and a guide RNA (gRNA), whose ~20-nucleotide spacer pairs with the genomic target and thereby determines cleavage specificity. The gRNA natively consists of a CRISPR RNA (crRNA) and a trans-activating crRNA (tracrRNA), which are commonly fused into a single-guide RNA (sgRNA) for genome-editing applications. Cleavage further requires a protospacer-adjacent motif (PAM; 5′-NGG-3′ for *Streptococcus pyogenes* Cas9) immediately downstream of the target, after which Cas9 generates a blunt DNA double-strand break (DSB). The break is repaired either by non-homologous end joining (NHEJ), which introduces small insertions or deletions, or by homology-directed repair when a donor template is supplied, the route exploited for targeted gene replacement [24]. As a revolutionary genome-editing tool, the CRISPR-Cas9 system has been widely adopted to overcome the inherently low efficiency of homologous recombination in fungi [25]. To date, this strategy has been successfully applied to a diverse array of filamentous fungi, including *Aspergillus niger* [26], *Fusarium oxysporum* [27], and *Ustilaginoidea virens* [28]. Notably, even for edible and medicinal fungi, which are notoriously recalcitrant to genetic manipulation due to their complex cell wall compositions, the CRISPR-Cas9 platform has demonstrated exceptional versatility and high editing efficiency, offering cutting-edge technical support for the genetic improvement of macrofungi [29,30,31].

Although PEG-mediated homologous recombination has enabled targeted gene disruption in *U. muhlenbergii*, its low efficiency and dependence on long homology arms still limit the throughput required for systematic functional genomics in this species. We hypothesized that Cas9-induced double-strand breaks at the target genomic locus would enhance homology-directed gene disruption in *U. muhlenbergii*, thereby allowing the use of shorter homology arms. To test this hypothesis, we established and evaluated a CRISPR-Cas9-assisted gene disruption system for this lichen-forming fungus. We developed a co-transformation strategy using a Cas9-sgRNA expression plasmid together with a selectable marker cassette carrying homology arms as short as 40 bp, and compared its disruption efficiency and specificity with those of conventional homologous recombination. As a proof of concept, we targeted six transcription factor genes selected as putative homologs of regulators controlling morphological transitions in *Saccharomyces cerevisiae* and *Candida albicans* and performed a preliminary phenotypic characterization of the resulting mutants. Overall, this genetic toolkit provides a functional framework for dissecting the regulatory networks that govern dimorphism in lichen-forming fungi and early fungal–photobiont interactions.

## 2. Materials and Methods

### 2.1. Strains and Culture Conditions

Wild-type strain *U. muhlenbergii* was initially isolated from a natural lichen thallus collected in Jilin Province, China [17], and was subsequently used as starting strain for generating the target mutants. Both wild-type and mutant strains were routinely cultured on Potato Dextrose Agar (PDA; BD Difco^TM^, BD Diagnostics, Sparks, MD, USA) plates at 22 °C.

### 2.2. Serial Dilution Assay

To compare the growth of the wild-type and mutant strains, all strains were cultured for 10 days, and the resulting fungal suspensions were adjusted to an OD_600_ of approximately 0.5. Each suspension was subjected to tenfold serial dilution to obtain undiluted 1, 10^−1^, 10^−2^, and 10^−3^ dilutions. Subsequently, 10 μL of each dilution was spotted onto PDA plates. After incubation for 10 days, colony growth and morphology were examined and photographed.

### 2.3. Identification of Transcription Factor Putative Homologs

Candidate transcription factor genes were identified by BLASTp searches against the *U. muhlenbergii* genome using *S. cerevisiae* and *C. albicans* protein sequences as queries. Reciprocal BLAST 2.17.0 searches against the NCBI non-redundant protein database were used to confirm the assignments; the best hits are summarized in Table 1.

### 2.4. Construction of the CRISPR-Cas9 Expression Vector

To develop a robust gene-editing platform for *U. muhlenbergii*, the Cas9 expression cassette was amplified using PCR from the template pCas9 [32]. This cassette comprises the Cas9 open reading frame under the transcriptional control of the transcription elongation factor (*tef*) promoter and the glucoamylase terminator. The resulting amplicon was directionally cloned into the pBS KS(+) vector between the *Eco*RI and *Sac*I restriction sites, yielding the intermediate plasmid pCas9-1.

To optimize sgRNA expression, we employed the endogenous Gln-tRNA promoter to drive its transcription [28]. A Gln-tRNA-RNA scaffold cassette, engineered with dual *Bsm*BI recognition sites for modular gRNA insertion, was synthesized by Sangon Biotech (Shanghai) Co., Ltd., Shanghai, China. and ligated into pCas9-1 at the *Kpn*I/*Eco*RI loci. The final construct, designated as pCas9-E63, serves as a universal CRISPR knockout backbone for all subsequent experiments.

### 2.5. Generation of Gene Replacement Constructs

Potential sgRNA spacers were designed using sgRNAcas9 (v3.0.5) [33]. The *U. muhlenbergii* genome was scanned on both strands in single-gRNA mode for 20-nt protospacers immediately upstream of a 5′-NGG-3′ PAM (N, any nucleotide), using the parameters -x 20 -l 40 -m 60 -o b -t s -n 5. Candidate spacers were then selected based on a GC content of 40–60%, the absence of four or more consecutive thymine residues, and a “Best” risk evaluation score, which indicates the lowest predicted off-target risk. Target sites were restricted to the 5′ region of the coding sequence, so that marker integration disrupts the coding sequence near its 5′ end and prevents the production of a partially functional protein. The sgRNA spacer sequences for the six target genes are listed in Table 2.

*Bsm*BI was used to digest the pCas9-E63 backbone as it cleaves outside its recognition sequence and generates two non-complementary 4-nt overhangs, allowing the annealed spacer oligonucleotides to be inserted directionally without introducing additional restriction sites into the spacer. Accordingly, 4 bp overhangs (5′-ACCT-3′ for the sense strand and 5′-AAAC-3′ for the antisense strand) were added to the 5′ termini of each oligonucleotide. The forward and reverse oligonucleotides were annealed to form double-stranded DNA inserts with cohesive ends and then ligated into the *Bsm*BI-linearized pCas9-E63 plasmid via T4 ligation, resulting in the precise integration of the sgRNA expression cassette. The sgRNA spacer sequences with 4 bp overhangs used for gene knockout in this study are listed in Supplementary Table S1.

### 2.6. Preparation of Donor DNA for CRISPR-Cas9-Mediated Gene Disruption

**Figure 1:**
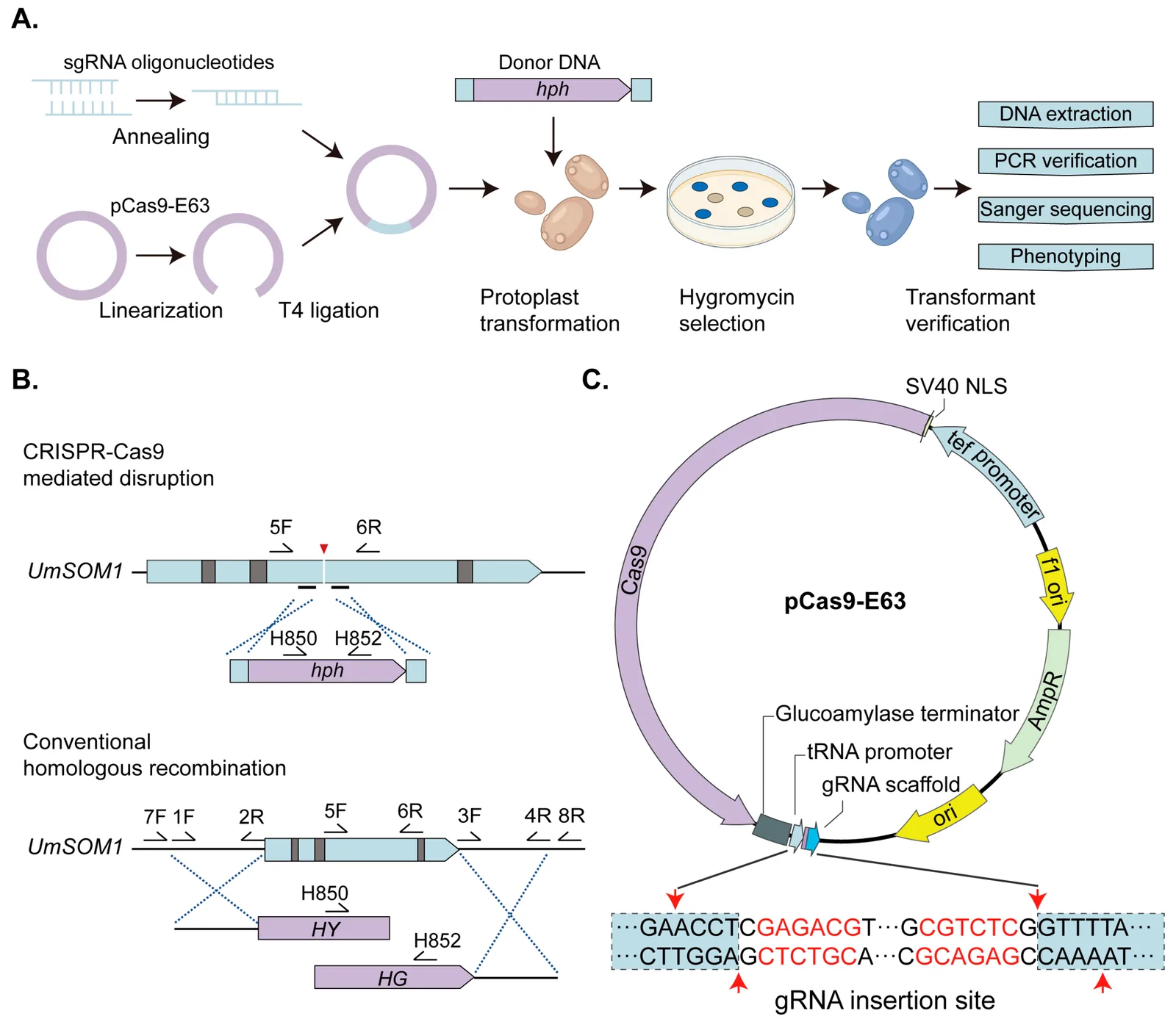
Schematic representation of the CRISPR-Cas9-mediated gene disruption strategy in *U. muhlenbergii.* (**A**) Overview of the experimental workflow. Annealed sgRNA spacer oligonucleotides were ligated into the BsmBI-linearized pCas9-E63 backbone using T4 DNA ligase. The resulting Cas9-sgRNA plasmid was co-transformed with the *hph* donor DNA into *U. muhlenbergii* protoplasts, and transformants were selected on hygromycin B-containing medium. Individual hygromycin-resistant colonies were isolated for genomic DNA extraction, PCR verification and Sanger sequencing, and subsequent phenotypic characterization. (**B**) Comparison of two strategies for *UmSOM1* gene disruption: (**Top**) CRISPR-Cas9-mediated double-strand break targeting the *UmSOM1* gene, and (**Bottom**) the conventional homologous recombination based gene knockout approach. (**C**) Schematic diagram of the CRISPR-Cas9 vector and the donor DNA repair template designed for targeted gene disruption. Red arrows indicate the BsmBI cleavage sites; sequences in red letters and blue boxes indicate the BsmBI recognition sites and micro-homology arms, respectively.

To facilitate the replacement of the target gene via homologous recombination, the hygromycin B phosphotransferase (*hph*) gene cassette was amplified from the pHYGT plasmid [34]. The primers were designed to incorporate 40 bp micro-homology arms at their 5′ termini (Supplementary Table S1). These flanking sequences are precisely homologous to the regions immediately upstream and downstream of the sgRNA-directed cleavage site (the 20 bp spacer region) within the target gene. An overview of the experimental workflow is shown in Figure 1A.

### 2.7. Preparation of Split-Marker DNA Fragments for UmSOM1 Disruption

The split-marker DNA fragments for *UmSOM1* disruption were prepared by overlapping PCR. Briefly, the 0.7 kb upstream and downstream flanking regions of *UmSOM1* (Figure 1B) were PCR-amplified with primer pairs 1F/2R and 3F/4R (Supplementary Table S2). These flanking sequences were then individually linked to the overlapping 5′ and 3′ regions of the *hph* cassette, which were amplified from pHYGT plasmid using primers HYG-F/HY-R and YG-F/HYG-R (Supplementary Table S2), respectively, resulting in two overlapping donor fragments ready for transformation.

### 2.8. Protoplast Preparation and Transformation

Protoplasts were prepared as described in previously published study [35,36], with the procedure briefly summarized as follows: Fresh yeast-form cells were harvested from 10-day-old PDA plates by washing with 1.2 M KCl and adjusted to an OD_600_ of 0.4–0.6. Cells were then subjected to an additional wash with 1.2 M KCl to enhance competency and collected by centrifugation at 3000 rpm for 5 min. The cell pellet was resuspended in 5 mL of digestion solution containing 25 mg/mL Driselase (Sigma-Aldrich) and incubated at 30 °C for 3 h with gentle shaking at 80 rpm. The resulting protoplasts were harvested and washed with STC buffer (20% *w/v* sucrose, 10 mM Tris-HCl, 40 mM CaCl_2_) and resuspended to final concentration of 10^7^ cells/mL.

For transformation, 500 ng of plasmid DNA and 1 µg of donor DNA fragment were mixed with 100 µL freshly prepared protoplast suspension and incubated at room temperature for 20 min before adding 1 mL PTC solution (40% PEG8000 in STC). After a further 20 min incubation, the transformation mixture was supplemented with 5 mL of Potato Dextrose Broth (PDB; BD Difco^TM^) medium containing 20% (*w*/*v*) sucrose. Following an additional 24 h incubation, the mixture was plated onto PDA plates containing 20% (*w*/*v*) sucrose and 30 µg/mL hygromycin B for selection. Hygromycin-resistant transformants were subsequently isolated and transferred to fresh PDA plates with hygromycin B for further experiments after 20–30 days of growth. All transformation experiments were performed in three independent biological replicates, and the numbers of transformants reported in the results represent the totals obtained across these replicates. Gene disruption efficiency was compared between the two approaches using a paired two-tailed Student’s *t*-test in GraphPad Prism 10.1.2, and *p* < 0.05 was considered statistically significant.

### 2.9. PCR-Based Verification of Transformants

Putative transformants were transferred to fresh selective PDA medium and cultured for genomic DNA extraction. The collected cells were resuspended in 600 µL 2 × CTAB extraction buffer and homogenized with 0.5 mm glass beads using the bead beater for 10 min. An equal amount of phenol/chloroform/isoamyl alcohol (25:24:1) was added to the lysate, followed by centrifugation. The aqueous phase was collected and genomic DNA was precipitated by adding 2 volumes of 100% ethanol. The resulting DNA pellet was air-dried and subsequently dissolved in ddH_2_O for PCR verification. Genomic DNA from the wild-type strain was included as a control.

For transformants generated using the CRISPR-Cas9 system, three primer pairs, 5F/6R, 5F/H852, and H850/6R (Supplementary Table S3), were used for PCR verification. The primer pair 5F/6R was used to amplify the entire target locus, whereas 5F/H852 and H850/6R were used to verify the 5′ and 3′ integration junctions of the donor DNA, respectively. For transformants generated by conventional homologous recombination, the primer pairs 7F/HY-R and YG-F/8R (Supplementary Table S2) were used to verify the 5′ and 3′ integration junctions, respectively. The primer pair 5F/6R (Supplementary Table S3) was used to determine whether the target gene remained in the genome, while H850/H852 was used to confirm the presence of the selectable marker cassette.

PCR amplification was performed using TaKaRa Taq^TM^ DNA Polymerase (Takara Bio Inc., Kusatsu, Japan) according to the manufacturer’s instructions. The resulting products were separated by agarose gel electrophoresis and compared with the expected fragment sizes. Transformants displaying the expected amplification patterns were considered putative gene-disruption mutants, and the relevant PCR products were subsequently subjected to Sanger sequencing to confirm correct integration.

### 2.10. PCR-Based Off-Target Mutation Analysis

Although sgRNA spacers were selected based on high specificity scores predicted by sgRNAcas9 software, the potential for off-target cleavage cannot be excluded due to the tolerance of 1–5 nucleotide mismatches between the sgRNA spacer and non-target genomic loci. To evaluate this possibility, putative off-target sites across the entire genome were predicted for each sgRNA (Supplementary Table S4). Specific primer pairs (Supplementary Table S5) were designed to amplify 300 to 500 bp flanking regions of each predicted off-target site. The resulting PCR products were subjected to Sanger sequencing, and the sequences were then aligned to the corresponding genomic regions to evaluate potential off-target mutations.

## 3. Results

### 3.1. A CRISPR-Cas9-Mediated Gene Disruption System Was Successfully Established in U. muhlenbergii

To optimize gene disruption in *U. muhlenbergii*, we developed a CRISPR-Cas9 mediated gene disruption system by integrating a Cas9 expression cassette with a native tRNA-driven sgRNA expression architecture, resulting in the construction the universal knockout vector pCas9-E63 (Figure 1C). The incorporation of tandem BsmBI restriction sites within the sgRNA scaffold enabled rapid and modular insertion of target-specific gRNA sequences. The integrity of the Cas9 expression cassette, Gln-tRNA promoter, and sgRNA scaffold was confirmed by Sanger sequencing prior to downstream gene disruption experiments.

### 3.2. CRISPR-Cas9-Mediated Disruption of UmSOM1 Is More Efficient than Conventional Homologous Recombination

Because the yeast-to-pseudohyphal transition is considered relevant to the early establishment of lichen symbiosis in *U. muhlenbergii*, we selected Um06c2280 as the pilot target for comparing CRISPR-Cas9-mediated gene disruption with conventional homologous recombination. This candidate was initially identified using *S. cerevisiae* Flo8, a well-characterized regulator of pseudohyphal and filamentous growth [37], as the query sequence. Although the overall sequence similarity to *S. cerevisiae* Flo8 was relatively low, Um06c2280 showed its strongest similarity to SomA of *Aspergillus fumigatus* (Table 1). SomA is the *A. fumigatus* homolog of the yeast Flo8 regulator and has been shown to control adhesion, asexual development, biofilm formation, and virulence in filamentous fungi [38]. Based on this best fungal hit and its functional relevance to fungal morphological development, we designated this gene *UmSOM1* and selected it as an initial locus for evaluating the CRISPR-Cas9 disruption strategy.

**Figure 2:**
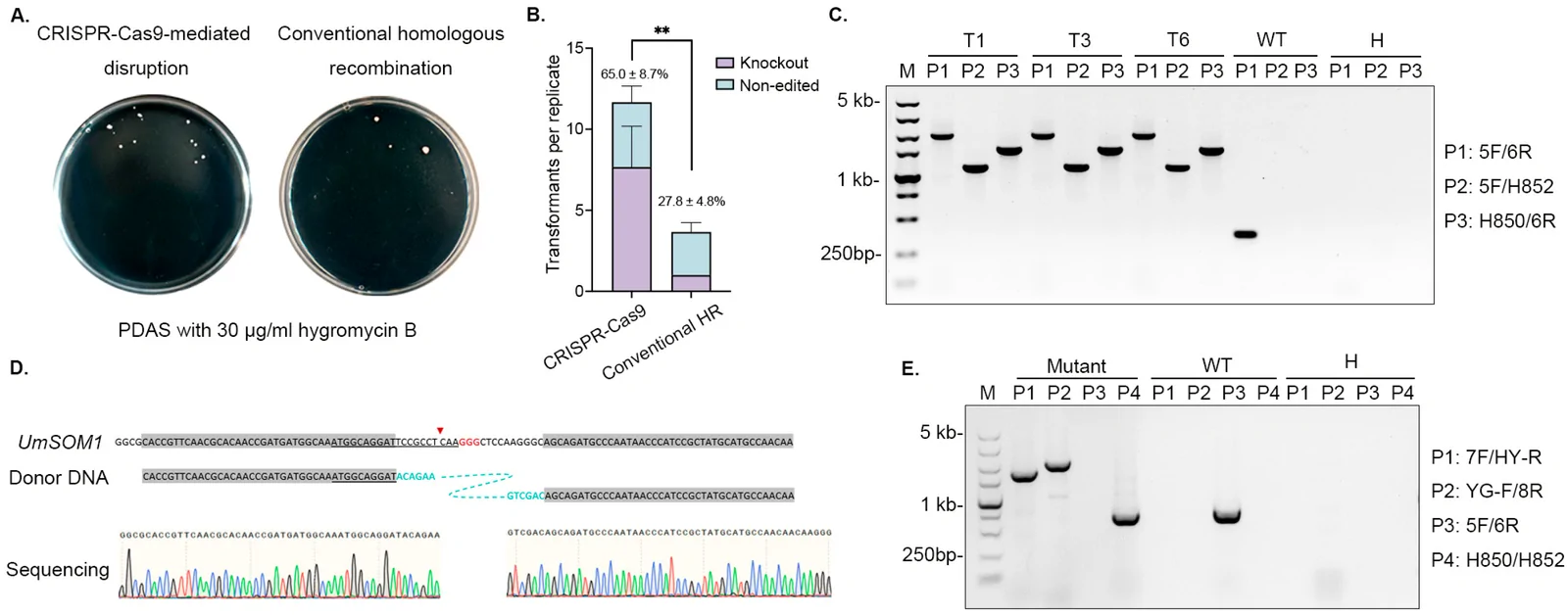
Comparison and molecular verification of CRISPR-Cas9-mediated gene disruption and conventional homologous recombination. (**A**) Representative images of transformant colonies growing on selective media obtained via CRISPR-Cas9 gene disruption (**left**) and conventional homologous recombination (**right**). (**B**) *UmSOM1* disruption efficiency using CRISPR-Cas9 versus conventional homologous recombination. Bars show mean transformants per replicate (*n* = 3), split into verified knockout mutants and non-edited transformants. Values above bars indicate the disruption efficiency. Data are presented as mean ± SD; ** *p* < 0.01 (paired two-tailed Student’s *t*-test). (**C**) PCR verification of the resulting transformants generated by the CRISPR-Cas9 method using gene-specific primers. T1, T3, and T6 are three independent transformants; WT and H indicate the wild-type and water controls for PCR, respectively. (**D**) Sanger sequencing confirmation of donor DNA integration at the *UmSOM1* locus. Grey shading indicates the homology arms of the donor DNA; the protospacer is underlined, the PAM is shown in red, and the red arrowhead indicates the Cas9 cleavage site. Cyan letters and dashed lines indicate the inserted hph sequence. (**E**) Diagnostic PCR verification of conventional homologous recombination transformants using flanking anchor primers to confirm correct donor DNA integration at the target locus.

Parallel gene disruption experiments were performed under strictly controlled conditions, with identical procedures for cell-wall digestion, protoplast regeneration, and recovery intervals. Each experiment was performed with three biological replicates. On selective media, the CRISPR-Cas9 approach yielded an average of 11 colonies per plate, resulting in 35 transformants in total, whereas the conventional homologous recombination approach yielded only 3 colonies per plate (11 transformants in total; Figure 2A). Subsequent PCR screening and sequencing confirmed 23 positive transformants, corresponding to a targeted gene disruption efficiency of 65.0%. In contrast, the homologous recombination approach exhibited substantially lower efficiency with 3 knockout transformants (27.8%) confirmed by sequencing, a significant reduction compared with the CRISPR-Cas9 method (*p* = 0.008, Figure 2B).

To verify the successful donor DNA integration, PCR screening was performed on the transformants using three primer pairs: 5F/6R, 5F/H852, and H850/6R (Supplementary Table S3). In the wild-type strain, amplification with the 5F/6R primer pair yielded a distinct 362 bp band, whereas no amplification was observed with the other two primer pairs (Figure 2C). In contrast, positive transformants exhibited a larger 2032 bp product when amplified with 5F/6R due to the insertion of the hygromycin resistance selection marker. Correct donor DNA integration in positive transformants was further confirmed by the expected diagnostic fragments of 1424 bp and 1721 bp amplified by the 5F/H852 and H850/6R primer pairs, respectively (Figure 2C). Detailed sequence alignment revealed that the integration sites of the donor DNA in the CRISPR-Cas9-mediated strains were exclusively restricted to the vicinity of the PAM (NGG) region; although the exact junctions varied slightly due to the inherent nature of end-joining repair, they were consistently located 3–5 bp adjacent to the NGG motif (Figure 2D).

In the conventional homologous recombination knockout transformants, the entire coding region of *UmSOM1* was replaced by the *hph* selection marker (Figure 1B); therefore, no PCR product was amplified from the positive transformants using the internal primer pair 5F/6R. Concurrently, successful integration at the correct locus was confirmed by the amplification of expected diagnostic bands using combinations of locus-flanking and marker-specific primers (i.e., the gene-upstream primer with the marker-downstream primer, and vice versa). These PCR profiles firmly demonstrated the precise disruption of the target gene (Figure 2E).

### 3.3. No Detectable Off-Target Effects Were Observed in CRISPR-Cas9-Generated Mutants

**Figure 3:**
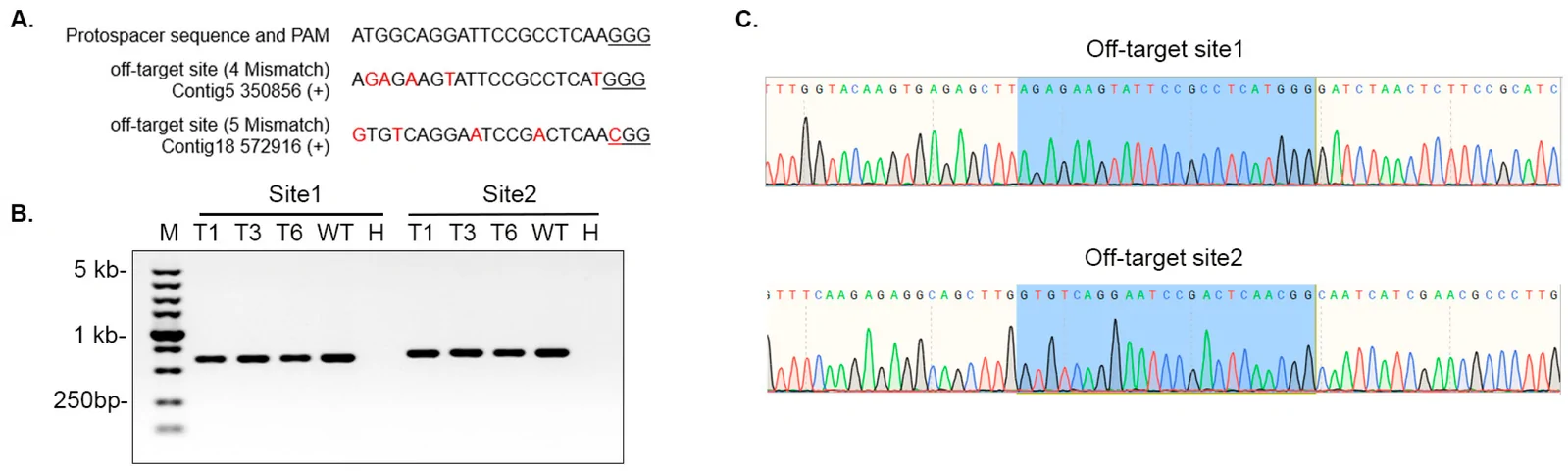
Off-target analysis of CRISPR-Cas9-mediated *UmSOM1* gene disruption transformants. (**A**) Sequence information of the two predicted potential off-target sites. Underlined sequences indicate the PAM, red letters indicate mismatches relative to the protospacer, and (+) indicates the sense strand of the genomic DNA. (**B**) Gel electrophoresis of PCR products amplifying the two potential off-target loci from randomly picked transformants (transformants 1, 3, and 6). (**C**) Sanger sequencing analysis of the two off-target loci, confirming the absence of insertions or mutations at these sites.

To assess the targeting specificity of the established CRISPR-Cas9 system in *U. muhlenbergii*, we performed a genome-wide prediction of potential off-target sites for the sgRNA targeting *UmSOM1*. The in silico analysis identified two potential off-target loci containing 4-mismatch and 5-mismatch sites, located at position + 350,856 on Contig5 and position + 572,916 on Contig18, respectively (Figure 3A). To experimentally evaluate potential non-specific cleavage events, locus-specific flanking primers for these two candidate regions were designed to amplify 400–500 bp target fragments. PCR amplification followed by Sanger sequencing revealed that the amplicons from three randomly selected knockout mutants exhibited identical band sizes to those of the wild-type strain (Figure 3B,C). Furthermore, sequence alignment revealed no detectable insertions, deletions, or nucleotide substitutions at either locus across all tested mutants. These results demonstrate the high fidelity of our CRISPR-Cas9-mediated gene disruption system in *U. muhlenbergii*, with no detectable off-target mutagenesis identified.

### 3.4. Five Additional Key Transcription Factor Genes Were Efficiently Disrupted and Verified

Following the successful disruption of *UmSOM1*, we applied the same strategy to five additional transcription factor genes to evaluate the versatility of the system across different loci. These genes were identified as putative homologs of regulators controlling morphological transition, stress adaptation, carbon metabolism and cell-cycle progression in *S. cerevisiae* and *C. albicans* (Phd1, Msn2, Mig1, Sfl1 and Mbp1) (Table 1).

**Figure 4:**
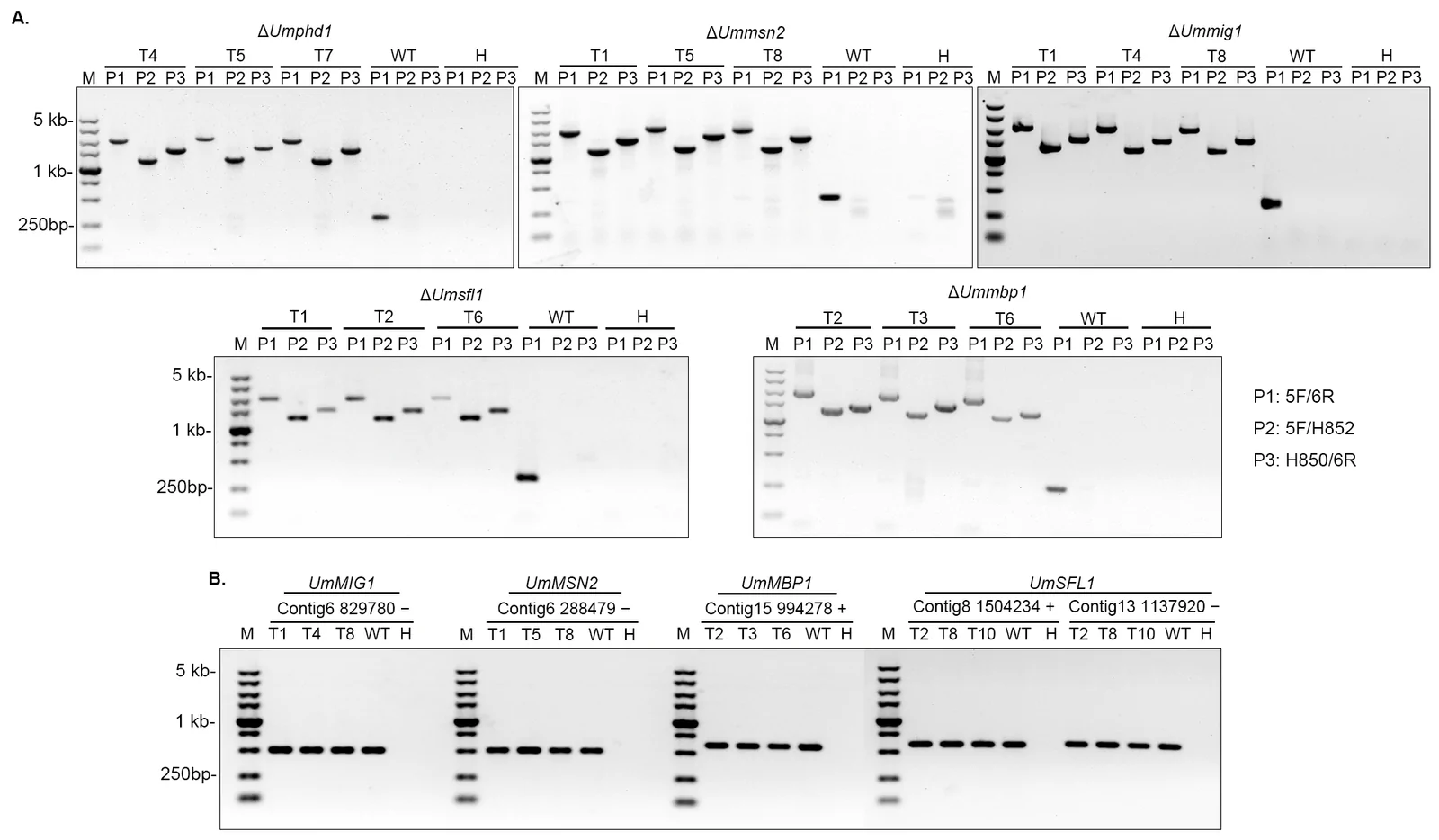
CRISPR-Cas9-mediated disruption and off-target analysis of five key fungal transcription factor homologs in *U. muhlenbergii*. (**A**) Diagnostic PCR validation of three independent mutants generated for each of the five target genes using gene-specific screening primers. (**B**) PCR-based off-target cleavage analysis at the predicted loci for the four genes. No potential off-target sites were predicted for *UmPHD1*, whereas the two predicted off-target loci for *UmSFL1* remained intact without non-specific cleavage.

By employing the aforementioned CRISPR-Cas9 transformation workflow, 14 to 35 transformants were randomly selected for each target gene and verified using PCR (Figure 4A). The results demonstrated that mutant strains for all five target genes were successfully constructed. The disruption efficiency (gene knockout rates) varied among the target loci, ranging from 34% to 64% (Table 3). Subsequently, three independent mutants for each gene were subjected to PCR amplification and Sanger sequencing of the predicted off-target sites (Supplementary Table S4), confirming the absence of detectable non-specific cleavage events (Figure 4B). These verified mutant strains were subsequently used for phenotypic characterization.

**Table 3:** Disruption efficiency of five target transcription factor genes *via* CRISPR-Cas9.

Target Gene	Total Transformants Screened	Verified Knockout Mutants	Disruption Efficiency (%)
*UmPHD1*	25	10	40.00%
*UmMSN2*	35	12	34.29%
*UmMIG1*	14	9	64.29%
*UmSFL1*	22	13	59.09%
*UmMBP1*	30	14	46.67%

### 3.5. Disruption of UmSOM1 Leads to Pseudohyphal Growth and Pigment Accumulation

**Figure 5:**
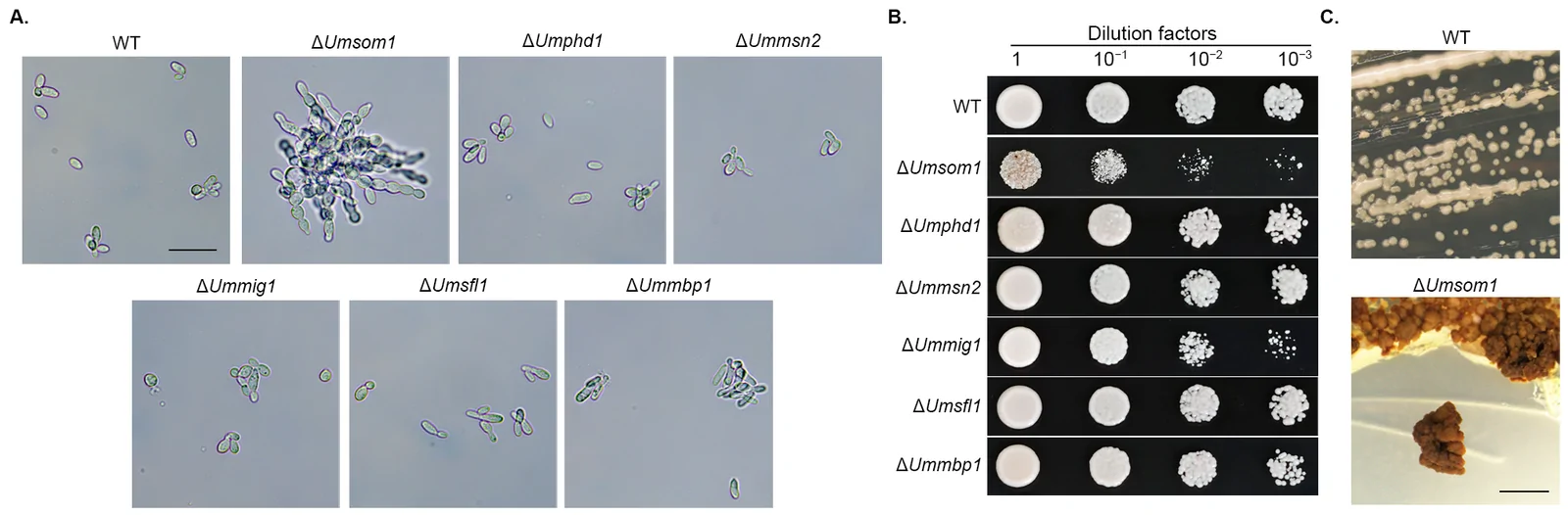
Disruption of UmSOM*1 *induced pseudohyphal growth and altered colony morphology in *U. muhlenbergii*. (**A**) Cellular morphology of WT and six transcription factor disruption mutants after 10 days of cultivation in PDB. WT and most mutants mainly showed yeast-like cells, whereas the *Umsom1* mutant formed clustered, elongated pseudohyphal cells. Scale bar = 20 μm. (**B**) Growth of the wild-type and mutant strains was assessed by serial dilution assay on PDA after 10 days of incubation. (**C**) Colony morphology of WT and the *Umsom1* mutant after 20 days of growth on PDA. Compared with the relatively smooth and pale WT colonies, the *Umsom1* mutant developed compact, granular, and rough colonies with strong brown pigmentation. Scale bar = 2 mm.

To compare the regulatory functions of different transcription factors on cellular morphology and colony development in *U. muhlenbergii*, we examined the cellular phenotypes of the wild-type strain and six transcription factor gene-disruption mutants after 10 days of cultivation in PDB. Microscopic observation revealed that the wild-type cells predominantly exhibited dispersed yeast-like or oval cellular morphology. Except for the *Umsom1* mutant, the *Umphd1, Ummsn2, Ummig1, Umsfl1, and Ummbp1* mutants generally maintained a yeast-form as their primary state (Figure 5A).

In the serial dilution assay, the *Umsom1* mutant showed substantially reduced growth compared with the wild-type strain. After 10 days of incubation, the mutant also exhibited a wrinkled colony morphology and yellowish pigment accumulation, indicating that disruption of *UmSOM1* affected both vegetative growth and colony morphology (Figure 5B). When the incubation period was extended to 20 days, the colony morphology of the *Umsom1* mutant became markedly altered, exhibiting compact, granular, and clumped structures with rough, uneven surfaces, together with pronounced accumulation of brown pigments (Figure 5C).

## 4. Discussion

Lichen-forming fungi are notoriously recalcitrant to genetic manipulation due to their slow growth and low transformation efficiency. Historically, tools such as ATMT and conventional homologous recombination have faced significant bottlenecks. Conventional homologous recombination based gene targeting relies on the endogenous homologous recombination machinery, including proteins of the Rad51-associated complex, and its efficiency is often limited by competition with the non-homologous end-joining (NHEJ) pathway [39,40,41]. To improve gene-targeting efficiency in genetically recalcitrant fungi, conventional homologous recombination based approaches often require relatively long flanking homology arms, typically 1–2 kb or longer, thereby increasing the complexity of donor DNA construction [42]. The biophysical rationale behind this cumbersome design is to augment the spatial scanning and docking probability of the recombinase complex, effectively forcing sequence alignment and search-and-match mechanics across the host chromatin. ATMT facilitates foreign DNA delivery, it predominantly leads to random genomic integration [43]. This unpredictable insertion behavior often triggers undesirable position effects, multi-copy concatenations, or unintended insertional mutagenesis of vital endogenous genes. Consequently, these traditional methodology suites are heavily constrained by laborious screening workflows and low reproducibility, failing to meet the demands for high-throughput, precise functional genomics. In this study, we successfully developed a CRISPR-Cas9 platform tailored for the lichen-forming fungus *U. muhlenbergii*. Remarkably, when targeting the key transcription factor UmSom1, this CRISPR-Cas9-based strategy achieved a recombination frequency of 65.0%, representing a more than twofold increase compared to conventional homologous recombination methods (Figure 2). Furthermore, this strategy yielded a substantially higher number of transformants. This remarkable improvement could be attributed to the simplified recombination mechanism; traditional large-fragment gene replacement typically requires three independent or sequential crossover events, whereas the CRISPR-Cas9 system significantly increases the insertion efficiency of the selection marker by generating site-specific DSBs, thereby bypassing the fundamental limitations of classical homologous recombination.

The length of homology arms required for precise gene targeting varies drastically across different fungal lineages. In the budding yeast *S. cerevisiae*, efficient targeted gene deletion can be achieved with homology sequences as short as 30–50 bp [44]. Within filamentous fungal systems, effective homologous recombination typically necessitates extensive homologous sequences spanning hundreds to thousands of base pairs [45,46]. Notably, our study demonstrates that applying the CRISPR-Cas9 system in *U. muhlenbergii* enables highly efficient gene disruption using short 40 bp homology arms flanking the selection marker. Compared to previously reported lichen-forming fungi gene disruption protocols [17], this short-homology approach completely eliminates the tedious and low-yield cloning steps required to ligate large-fragment flanking arms to the selection gene, greatly streamlining the vector construction and donor DNA preparation workflows.

High editing fidelity is a prerequisite for accurate functional genomics. Using the optimized CRISPR-Cas9 workflow, we efficiently generated three independent gene-disruption mutants for each of six transcription factor genes, yielding a total of 18 mutant strains. No potential off-target sites were predicted for the sgRNA targeting UmPHD1 during in silico spacer design. For the other five target genes, all predicted off-target sites were examined in 15 independent mutants, and no off-target mutations were detected at any of these sites, demonstrating the high specificity of the CRISPR-Cas9 system.

The six genes targeted in this study encode putative homologs of transcriptional regulators originally characterized in *S. cerevisiae* (Flo8, Phd1, Msn2, and Mig1) or *C. albicans* (Sfl1 and Mbp1). In these model yeasts, these regulators collectively participate in diverse biological processes, including filamentous growth and morphogenesis, stress adaptation, carbon metabolism, and cell-cycle regulation [37,47,48,49,50,51]. To investigate whether these functions are conserved in the lichen-forming fungus *U. muhlenbergii*, we systematically compared the growth, colony morphology, pigmentation, and hyphal morphology of three independent disruption mutants for each target gene with those of the wild-type strain. Among the six mutant groups, the Δ*Umsom1* mutants exhibited the most pronounced phenotypic alterations.

As a classic downstream target of the cAMP-PKA pathway, Flo8 dictates diverse growth phenotypes by modulating specific target genes. In *S. cerevisiae*, Flo8 primarily activates *FLO11/MUC1* expression to mediate cell–cell adhesion, invasive growth, flocculation, and pseudohyphal formation [52]; notably, the lack of these phenotypes in the standard laboratory strain S288C is attributed to a natural mutation in *FLO8*, whereas its overexpression significantly enhances these traits [37,53]. This regulatory mechanism is highly conserved in opportunistic human pathogens and directly linked to pathogenicity. In *C. albicans*, Flo8 is indispensable for hyphal development and virulence, as *flo8* deletion abolishes hyphal morphogenesis and obliterates systemic virulence in murine models [54,55]. Similarly, in *A. fumigatus*, the homologs somA (containing a conserved LUFS/LisH-like domain) can partially rescue *S. cerevisiae flo8* mutants heterologously. However, inside the native *A. fumigatus* system, *somA* deficiency impairs vegetative growth, blocks asexual development, and compromises adherence, biofilm formation, and virulence [38]. Together, these findings highlight the Flo8/SomA family as a class of conserved, pleiotropic transcriptional regulators linking fungal morphogenesis to pathogenesis.

Interestingly, disruption of the Flo8 homolog in *U. muhlenbergii* resulted in constitutive pseudohyphal growth and strong brown pigmentation even in the absence of external inducing cues, accompanied by reduced growth (Figure 5). This phenotype differs from the canonical role of Flo8 in *S. cerevisiae*, where Flo8 acts mainly as a cAMP-PKA dependent activator of *FLO11* and promotes adhesion, invasive growth and pseudohyphal differentiation. However, in the lichenized fungus *U. muhlenbergii*, elevated cAMP signaling has been shown to induce yeast-to-pseudohyphal transition and heavily melanized, lichen cortex-like structures [17]. Thus, the phenotype of the Flo8-homolog knockout resembles a constitutively induced cAMP-like developmental state. One possible explanation is that the Flo8 homolog in *U. muhlenbergii* has acquired a regulatory role in maintaining yeast-like growth or in restraining inappropriate activation of cAMP-responsive morphogenetic and melanization programs. Loss of this regulator may release pseudohyphal development and pigment biosynthesis from normal control, while imposing a fitness cost due to excessive cell wall remodeling, polarized growth and pigment production.

Because lichen formation involves multiple partners, lichen-forming fungi are likely to require a complex array of extracellular receptors, partner recognition molecules, and signalling components that mediate growth and morphological change, the establishment of the symbiotic interface, and protection against non-symbiotic competitors. They may in particular have evolved expanded families of partner-specific sensing genes that allow them to detect and pair with compatible algae. So far, however, cAMP-PKA signalling and the Ump1 MAP kinase pathway are the only pathways shown to be required for the yeast-to-pseudohyphal transition and for symbiotic interaction with algal cells in *U. muhlenbergii* [17,18], and the kinases and transcription factors acting downstream of them remain unidentified. As *U. muhlenbergii* is currently the only lichen-forming fungus in which targeted gene disruption has been established, the efficient gene disruption system reported here should accelerate the identification of these components. Beyond their value as models for symbiosis, lichens are also a substantial source of structurally diverse secondary metabolites with pharmacological potential. Because sequential and multiple gene disruption are now readily achievable, this platform also provides a practical route to the functional characterization of biosynthetic gene clusters in *U. muhlenbergii*.

## 5. Conclusions

In conclusion, we established an efficient and highly specific CRISPR-Cas9-mediated gene disruption system in the lichen-forming fungus *U. muhlenbergii*. Its successful application to six transcription factor genes demonstrates its robustness and broad applicability for targeted genetic manipulation. Moreover, phenotypic characterization of the *Umsom1* mutants revealed a potential role for *UmSOM1* in vegetative growth, morphological development, and pigment accumulation, highlighting the utility of this system for functional gene analysis. The high gene disruption efficiency and absence of detectable mutations at the predicted off-target sites provide a solid foundation for the future construction of arrayed CRISPR mutant libraries in *U. muhlenbergii*. Such resources will enable systematic functional genomic screening of candidate regulators involved in fungal–algal symbiosis, transcription factor networks, morphological transitions, environmental stress adaption, and specialized metabolism in lichen-forming fungi. More broadly, this genetic toolkit will facilitate experimental dissection of conserved and lineage-specific genes that may have contributed to the evolution and diversification of lichen-forming fungi.

## Figures and Tables

**Table 1:** Identification of putative transcription factor homologs in *U. muhlenbergii* using query sequences from *S. cerevisiae* and *C. albicans*.

Model Query	*U.m* Target and NCBI No.	E-Value (to Um)	Identity (%)	Best Fungal Hit (Species)	E-Value (to Fungi)
Flo8 *S. cerevisiae*	UmSom1 Um06c2280	4.73 × 10^−5^	25%	somA *A. fumigatus*	8.35 × 10^−158^
Phd1 *S. cerevisiae*	UmPhd1 Um04c5810	2.49 × 10^−48^	67.30%	stuA *A. flavus*	0
Msn2 *S. cerevisiae*	UmMsn2 Um03c4960	1.23 × 10^−17^	63%	sebA *A. fumigatus*	1.50 × 10^−129^
Mig1 *S. cerevisiae*	UmMig1 Um14c4440	3.18 × 10^−29^	66.20%	creA *A. flavus*	2.06 × 10^−156^
Sfl1 *C. albicans*	UmSfl1 Um01c1870	1.98 × 10^−32^	51.80%	Sfl1 *C. albicans*	1.98 × 10^−32^
Mbp1 *C. albicans*	UmMbp1 Um17c0920	3.26 × 10^−39^	55.20%	res1 *S. pombe*	3.94 × 10^−75^

**Table 2:** Details of sgRNA sequences and predicted off-target profiles for the targeted genes. 1 M–5 M indicate the number of predicted off-target sites carrying one to five mismatches relative to the protospacer.

Protein Name	Protospacer + PAM(5′-3′)	GC%	1 M	2 M	3 M	4 M	5 M	Total Off-Target
UmSom1	ATGGCAGGATTCCGCCTCAAGGG	55%	0	0	0	1	1	2
UmPhd1	TCGGACCAATGCATCTCAAGGGG	50%	0	0	0	0	0	0
UmMig1	CAGCCTACGCCTACTCACAAGGG	55%	0	0	0	0	1	1
UmMsn2	CTCTCCCTACATTGTGCTCAGGG	50%	0	0	0	0	1	1
UmSfl1	AAAACTAGTGTGTCACAACCAGG	40%	0	0	1	0	1	2
UmMbp1	GGGTACAATCTTTCACCATGCGG	45%	0	0	0	1	0	1

## Data Availability

The nucleotide sequences of the six genes analyzed in this study have been deposited in the NCBI GenBank database under accession numbers PZ714560 (UmSom1), PZ714561 (UmPhd1), PZ714562 (UmMsn2), PZ714563 (UmMig1), PZ714564 (UmSfl1), and PZ714565 (UmMbp1). All other data supporting the findings of this study are included in the article and its Supplementary Materials.

## Supplementary Materials

The following supporting information can be downloaded at https://www.mdpi.com/article/10.3390/jof12080622/s1: Table S1: Primers used for the construction and amplification of donor DNA cassettes in this study; Table S2: Primers used for *UmSOM1* knockout using traditional homologous recombination; Table S3: Primers used for the PCR verification of target gene disruption in transformants; Table S4: Detailed sequences and genomic loci of predicted off-target sites for the designed sgRNAs; Table S5: Primers used for the PCR amplification and sequencing of predicted off-target sites.

## Abbreviations

The following abbreviations are used in this manuscript:

ATMT	Agrobacterium tumefaciens-mediated transformation
T-DNA	Transfer DNA
DSB	DNA double-strand break
PDA	Potato Dextrose Agar
PDB	Potato Dextrose Broth
PEG	Polyethylene glycol
PAM	Protospacer adjacent motif
*hph*	Hygromycin B phosphotransferase (*hph*)
*tef*	Transcription elongation factor (*tef*)
NHEJ	Non-homologous end-joining
